# Large-scale survey for novel genotypes of *Plasmodium falciparum *chloroquine-resistance gene *pfcrt*

**DOI:** 10.1186/1475-2875-11-92

**Published:** 2012-03-28

**Authors:** Nobuyuki Takahashi, Kazuyuki Tanabe, Takahiro Tsukahara, Mawuli Dzodzomenyo, Lek Dysoley, Boualam Khamlome, Jetsumon Sattabongkot, Masatoshi Nakamura, Miki Sakurai, Jun Kobayashi, Akira Kaneko, Hiroyoshi Endo, Francis Hombhanje, Takafumi Tsuboi, Toshihiro Mita

**Affiliations:** 1 Department of International Affairs and Tropical Medicine, Tokyo Women's Medical University School of Medicine, 8-1 Kawada-cho, Shinjuku-ku, Tokyo 162-8666, Japan; 2 Department of Molecular Protozoology, Research Institute for Microbial Diseases, Osaka University, Suita, Osaka, Japan; 3 Department of Biological, Environmental and Occupational Health Sciences, School of Public Health, University of Ghana, Legon, Ghana; 4 National Center For Parasitology Entomology and Malaria Control, Phnom Pehn, Cambodia; 5 Prevention Division, Hygiene Prevention Department, Ministry of Health, Vientiane, Lao P.D.R; 6 Department of Entomology, Armed Forces Research Institute of Medical Sciences, Bangkok, Thailand; 7 Department of Tropical Medicine and Parasitology, Dokkyo Medical University, Tochigi, Japan; 8 Graduate School of International Health Development, Nagasaki University, Nagasaki, Japan; 9 Department of Microbiology, Tumor and Cell Biology, Karolinska Institutet, Stockholm, Sweden; 10 Department of Parasitology, Osaka City University Graduate School of Medicine, Osaka, Japan; 11 Institute of Tropical Medicine, Nagasaki University, Nagasaki, Japan; 12 Center for Health Research, Divine Word University, Madang, Papua New Guinea; 13 Cell-free Science and Technology Research Center, and Venture Business Laboratory, Ehime University, Matsuyama, Ehime, Japan

**Keywords:** *Plasmodium falciparum*, Chloroquine resistance, *pfcrt*, Microsatellite, Haplotype network, Evolution

## Abstract

**Background:**

In *Plasmodium falciparum*, resistance to chloroquine (CQ) is conferred by a K to T mutation at amino acid position 76 (K76T) in the *P. falciparum *CQ transporter (PfCRT). To date, at least 15 *pfcrt *genotypes, which are represented by combinations of five amino acids at positions 72-76, have been described in field isolates from various endemic regions. To identify novel mutant *pfcrt *genotypes and to reveal the genetic relatedness of *pfcrt *genotypes, a large-scale survey over a wide geographic area was performed.

**Methods:**

Sequences for exon 2 in *pfcrt*, including known polymorphic sites at amino acid positions 72, 74, 75 and 76, were obtained from 256 *P. falciparum *isolates collected from eight endemic countries in Asia (Bangladesh, Cambodia, Lao P.D.R., the Philippines and Thailand), Melanesia (Papua New Guinea and Vanuatu) and Africa (Ghana). A haplotype network was constructed based on six microsatellite markers located -29 kb to 24 kb from *pfcrt *in order to examine the genetic relatedness among mutant *pfcrt *genotypes.

**Results:**

In addition to wild type (CVMNK at positions 72-76), four mutant *pfcrt *were identified; CVIET, CVIDT, SVMNT and CVMNT (mutated amino acids underlined). Haplotype network revealed that there were only three mutant *pfcrt *lineages, originating in Indochina, Philippines and Melanesia. Importantly, the Indochina lineage contained two mutant *pfcrt *genotypes, CVIET (n = 95) and CVIDT (n = 14), indicating that CVIDT shares a common origin with CVIET. Similarly, one major haplotype in the Melanesian lineage contained two *pfcrt *genotypes; SVMNT (n = 71) and CVMNT (n = 3). In Africa, all mutant *pfcrt *genotypes were the CVIET of the Indochina lineage, probably resulting from the intercontinental migration of CQ resistance from Southeast Asia.

**Conclusions:**

The number of CQ-mutant lineages observed in this study was identical to that found in previous studies. This supports the hypothesis that the emergence of novel CQ resistance is rare. However, in the mutant *pfcrt *genotypes, amino acid changes at positions 72, 74 and 75 appear to have recently been generated at least several times, producing distinct *pfcrt *mutant genotypes. The occurrence of new mutations flanking K76T may yield stronger resistance to CQ and/or a higher fitness than the original *pfcrt *mutant.

## Background

The spread of drug-resistant *Plasmodium falciparum*, the most virulent malaria parasite, represents a serious concern for the treatment and control of falciparum malaria. It is generally believed that the emergence of drug-resistant *P. falciparum *is rare and geographically restricted [1-4]. Clinical resistance to chloroquine (CQ) was first identified simultaneously in two different geographic regions in the late 1950s; Southeast Asia (Thailand-Cambodia border) [5] and South America [6]. CQ resistance then expanded to neighbouring countries in the 1960s, and nearly all Southeast Asian countries by the mid-1970s [4]. In Melanesia, resistance to CQ was reported in the early 1960s in Indonesian West Papua, shortly after mass administration of CQ in medicated table salt [7]. Subsequently, it spread to Papua New Guinea [8] and the Solomon Islands [4] in 1976 and 1980, respectively. In Africa, CQ resistance was first reported in the late 1970s in Tanzania [9,10], and it was found to be have been introduced from Southeast Asia [11].

Since the discovery of *P. falciparum *chloroquine transporter (PfCRT) as a primary target of CQ resistance [12], reports on the geographic origins and spread of CQ-resistant *P. falciparum *have accumulated [1]. PfCRT is localized to the parasite food vacuole and is known to have > 10 polymorphic amino acid sites [12]. Among these, an amino acid change from Lys (K) to Thr (T) at position 76 (K76T) plays a decisive role in conferring resistance to CQ [12]; the mutation greatly reduces the accumulation of CQ in the parasite food vacuole by accelerating efflux of CQ [13]. Microsatellite (MS) analysis flanking the PfCRT gene, *pfcrt*, has revealed that the geographic origin of CQ resistance is quite limited, with only four CQ resistant lineages initially identified: one in Indochina/Africa, one in Melanesia, and two in South America (Brazil/Peru and Ecuador/Colombia) [1]. Subsequently, one distinct CQ-resistant lineage was discovered in isolates originating in the Philippines [14].

These CQ resistant lineages harbour one of four mutant *pfcrt *genotypes at positions 72-76 (CVIET, SVMNT, CVMNT and CVMET; mutations underlined), with the SVMNT genotype being found in Brazil/Peru and Melanesia lineages [1,14]. In addition, at least 10 mutant *pfcrt *genotypes have recently been identified in field isolates from various endemic regions; SVMIT (Guyana [15]), SVMET (Colombia [16]), SVIET (Indonesian Papua [17]), SVMDT (Philippines [18]), CVMET (Colombia [16]), CVMNN (Indonesia [19]), CVTNT (Cambodia [20]), CVIDT (Madagascar [21], India [22], Cambodia [20]), CVMDT (Philippines [18]) and RVMNT (Guyana [15]). Meanwhile, recovery of CQ sensitivity was reported after the use of CQ was abandoned in Malawi [23-25]. Similar recovery has also been reported on Hainan Island, China [26]. This recovery was thought to be due to the re-introduction of susceptible parasites harbouring a CQ-sensitive *pfcrt *[27,28], but a back mutation in *pfcrt *at position 76 from the resistant-type amino acid (T) to the sensitive-type amino acid (K) may also be potentially involved in the recovery of CQ sensitivity in some endemic areas, although this has yet to be confirmed.

This possibility can be inferred from the previous finding of a laboratory-maintained CQ-sensitive *P. falciparum *clone (106/1) originating in Sudan that apparently underwent a back mutation from T to K at position 76 [12]. It is thus likely that novel *pfcrt *genotypes may be identified through surveys of large numbers of *P. falciparum *isolates from diverse geographic areas. In support of this, recent large-scale surveys of genotypes of the dihydrofolate reductase gene *dhfr*, a target of pyrimethamine, and of the dihydropteroate gene *dhps*, a target of sulphadoxine, have identified several novel genotypes from wide geographic areas [24,25], and have provided insights into the evolutionary history of drug resistance in *P. falciparum*.

In this study, genotyping of *pfcrt *and haplotyping of MS markers flanking *pfcrt *were performed in *P. falciparum *isolates collected from Asia (Bangladesh, Cambodia, Lao P.D.R., Philippines and Thailand), Melanesia (Papua New Guinea and Vanuatu), and Africa (Ghana). Results showed that there were only three CQ-resistant lineages, all of which were identified previously [1,14]. No evidence of back mutation was observed at position 76. Importantly, however, among *pfcrt *genotypes having the K76T mutation, amino acid changes other than K76T appear to have been recently generated on at least several occasions, producing novel *pfcrt *mutant genotypes.

## Methods

### Study area and samples

Blood samples were obtained from *P. falciparum*-infected individuals living in eight malaria endemic countries as follows.

(1) Thailand: isolates were obtained during a longitudinal study on malaria transmission at a village in Kanchanaburi Province located at the western border of Thailand. Pre-treatment venous blood from a falciparum malaria-positive villager was obtained for this study between 2000 and 2003.

(2) Lao P.D.R.: isolates were specifically obtained for this study. Finger-pricked blood samples were taken in Napong Village, Boulapha District, Khammouane Province, in March 1999.

(3) Cambodia: isolates were obtained from finger-prick blood samples taken in Chumkiri District, located in the southeastern coastal Province of Kampot, in December 2004 [29].

(4) Bangladesh: isolates were obtained from finger-prick blood samples taken in Bandarban district hospital from October to December 2007 and six malaria endemic villages; Sultanpur, Chemidalupara, Kyaching ghata natun para, Saingya daneshpara, Faruqpara and Empupara, in Bandarban District, in March 2008 [30].

(5) Philippines: isolates were obtained from pre-treatment venous blood samples taken on Palawan Island, Palawan Province, in May and October 1997 [31].

(6) Papua New Guinea: isolates were obtained from finger-prick blood samples taken during *in vitro *studies at Wewak General Hospital located in the Wewak District, East Sepik Province, in 2002 and 2003 [32].

(7) Vanuatu: isolates were obtained from finger-prick blood samples taken on Gaua Island, in February 1997, Pentecost Island, in February 1998 [33].

(8) Ghana: isolates were obtained from finger-prick blood samples taken from three villages, Okyereko, Mpota and Apam, near Winneba, a western coastal region, in November 2004 [4].

### Ethical considerations

Before enrolment, written informed consent was obtained from all study subjects. In the case of children, consent was obtained from a parent or legal guardian. This study was approved by (1) The Institutional Ethics Committee of the Thai Ministry of Public Health, the Human Subjects Research Review Board of the United States Army, (2) The Center of Malariology, Parasitology and Entomology (CMPE), Lao P.D.R., the Research Committee of the Ministry of Public Health (MoPH), Lao P.D.R., (3) The National Center for Parasitology, Entomology and Malaria Control (CNM), Cambodia, (4) The Bangladesh Medical Research Council and the local regulatory body of health in Bandarban, Bangladesh, (5) The Palawan Provincial Health Office, Philippines, (6) The National Department of Health Medical Research Advisory Committee of Papua New Guinea, (7) The Vanuatu Department of Health, Vanuatu, and (8) The Ministry of Health/Ghana Health Service, Ghana.

### DNA extraction

Finger-prick blood was spotted onto chromatography filter paper ET31CHR (Whatman, Maidstone, UK) in Lao P.D.R., Cambodia, Bangladesh, Papua New Guinea, Vanuatu and Ghana. In Thailand and the Philippines, venous blood was transferred into heparin-containing test tubes. Parasite DNA was extracted using QIAamp DNA mini kits (Qiagen, Hilden, Germany) from a quarter of a dried blood spot or a corresponding amount of blood (25 μl), in accordance with the manufacturer's instructions. The Lao P.D.R. and Bangladesh samples were extracted using QIAamp DNA mini kits with the QIAcube™ (Qiagen) tissue protocol.

### *pfcrt *genotyping

The *pfcrt *gene was amplified by nested PCR using two sets of primers designed to amplify a region of exon 2 including known polymorphic sites at amino acid positions 72, 74, 75 and 76. PCR primers were designed after the sequence of 3D7 clone [GenBank: NC_004328]. Primer sequences and PCR conditions are shown in Additional file 1. Amplified product (aa 57-120) was purified using ExoSAP-IT (GE Healthcare UK Ltd., Buckinghamshire, UK) and were directly sequenced with a DYEnamic ET terminator kit in the MegaBACE 1000 DNA sequencer (GE Healthcare UK Ltd.). Data on *pfcrt *genotypes previously obtained from Papua New Guinea were included in the present analysis [32].

### Microsatellite haplotyping

Variations in the number of TA repeats located at 0.59 kb, 10.389 kb, 23.576 kb, -2.814 kb and -29.268 kb in *pfcrt *and ATT repeats located at 10.389 kb in *pfcrt *were measured using PCR protocol previously described with some modifications (Additional file 2) [34]. Briefly, each MS marker was amplified by semi-nested PCR using fluorescent 5'-end labeled primers (Applied Biosystems., Foster city, CA, USA) in an ABI 2720 thermal cycler (Applied Biosystems). Amplified products were analysed using an ABI 377 DNA sequencer and GeneScan 3.1.2 software with GENESCAN™ 400 HD ROX size standard (Applied Biosystems), followed by size determination using a Genotyper 2.0 (Applied Biosystems). When two or more polymorphisms were detected, these isolates were considered to be mixed infections and excluded them from further analysis. As it has been well documented that wild-type parasites show extensive MS variations due to the lack of selective CQ sweeps [1], MS haplotypes were determined only for *pfcrt *mutant isolates.

### Phylogenetic analysis

In order to assess the genetic relationships among CQ-resistant *pfcrt *genotypes, a median-joining haplotype network was constructed based on alleles at the six MS loci using the Network 4.6 software [35]. Median joining is a method for constructing genetic networks to identify the minimum spanning tree by favouring short connections [35].

## Results

### *pfcrt *genotypes

**Figure 1:**
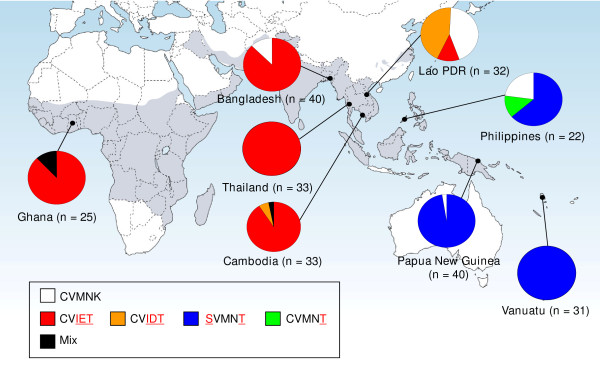
**Geographical distribution of *pfcrt *genotypes in 256 *Plasmodium falciparum *isolates from eight countries in Asia, Africa and Melanesia**. Capital letters in the box denote amino acid residues at positions 72, 73, 74, 75 and 76, with mutations identified in red. Mixed genotypes include combinations of CVIE/DT (n = 1), CVMNK/T (n = 1), CVIN/ET (n = 1) and CVIEK/T (n = 1). Gray colour shows a malaria endemic area.

Among 263 *P. falciparum *isolates examined in this study, *pfcrt *genotypes were successfully determined in 256 samples. In addition to the wild-type CVMNK (10%), four *pfcrt *mutant genotypes were identified; CVIET (48%), CVIDT (6%), SVMNT (33%) and CVMNT (1%). Mixed genotypes were observed in four samples (2%) (Figure 1). In endemic regions of Indochina (Thai and Cambodia), South Asia (Bangladesh) and Africa (Ghana), CVIET was the predominant mutant genotype, which is consistent with previous studies [1,11]. One exception was Lao P.D.R., where the wild genotype and CVIDT were equally predominant, with CVIET showing a low frequency. In the Philippines, as previously observed [14], SVMNT was the dominant mutant genotype, and CVMNT was also found. In Melanesia, only SVMNT was found as a mutant genotype, as reported previously [36]. We did not observe any novel mutations at amino acid positions 57-120, other than those known at positions 72, 74, 75 and 76.

### Microsatellite haplotypes and network analysis

**Figure 2:**
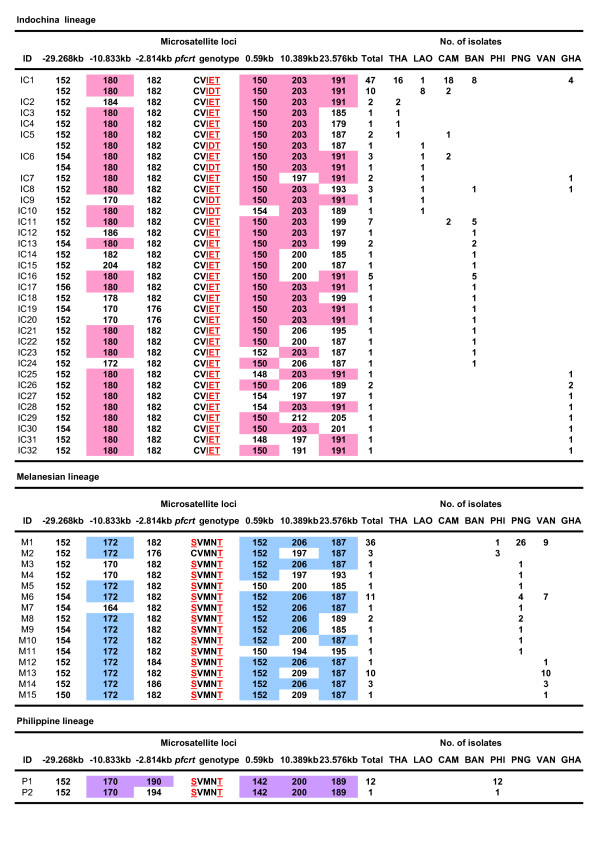
**Microsatellite haplotypes found in 196 chloroquine-resistant *P. falciparum *isolates from eight countries in Asia, Africa and Melanesia**. The number of TA repeats at each MS locus is shown. MS alleles in the most frequent haplotype are shown in pink (Indochina lineage), blue (Melanesian lineage) and violet (Philippine lineage). THI; Thailand, LAO; Lao P.D.R., CAM; Cambodia, BAN; Bangladesh, PHI; Philippines, PNG; Papua New Guinea, VAN; Vanuatu, GHA; Ghana.

**Figure 3:**
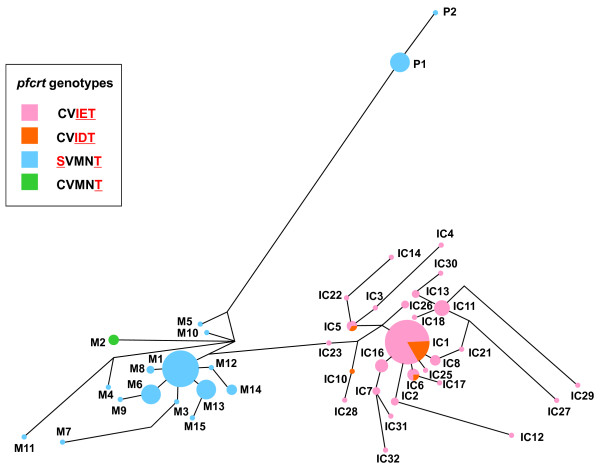
**Haplotype network diagram in *Plasmodium falciparum *isolates harbouring *pfcrt *mutation**. Network tree is shown according to *pfcrt *genotypes. The haplotype network was constructed for 196 *P. falciparum *isolates harbouring *pfcrt *mutation comprises 49 microsatellite haplotypes, based on allelic variations in six microsatellite loci flanking the *pfcrt *locus (see text for details). The size of each circle corresponds to the number of samples sharing the same haplotype, and the length of an edge is proportional to a variation in repeat number between two haplotypes. IC; Indochina lineage, M; Melanesian lineage, P; Philippine lineage.

Among 227 isolates harbouring *pfcrt *mutant genotypes, 196 samples were successfully determined for MS haplotypes. In total, 49 MS haplotypes were identified, including the three previously known major haplotypes (IC1, M1 and P1) and those haplotypes similar to IC1, M1 or P1 (Figure 2). To assess the genetic relatedness of *pfcrt *mutants, a haplotype network based on size variations at all MS markers was constructed. The haplotype network clearly showed three distinct clusters of *pfcrt *mutants (Figure 3). The first lineage (Indochina lineage) consisted of the most prevalent haplotype IC1 (n = 57), having a combination of alleles of 152-180-182-150-203-191 at the MS loci of -29.268 kb, -10.833 kb, -2.814 kb, 0.59 kb, 10.389 kb and 23.576 kb, respectively, and those haplotypes closely related to IC1. Nearly all isolates in this lineage harboured the CVIET genotype. However, several isolates (IC1, IC5, IC6, IC9 and IC10; n = 14) contained the CVIDT genotype. This indicates that this mutant genotype shares a common origin with CVIET, and that MS haplotype IC1 contained two mutant *pfcrt *genotypes. The second lineage (Melanesian lineage) contained the most prevalent haplotype M1 (n = 36), having an allele combination of 152-172-182-152-206-187 and those haplotypes related to M1. All but M2 (CVMNT, n = 3) harboured the SVMNT genotype. This indicates that MS haplotype M1 also contained two mutant *pfcrt *genotypes. In the third lineage (Philippine lineage), all but one (P2) harboured an allele combination of 152-170-190-142-200-189 (P1). As previously observed in the Melanesian lineage [1], all isolates in the Philippine lineage harboured the SVMNT genotype.

**Figure 4:**
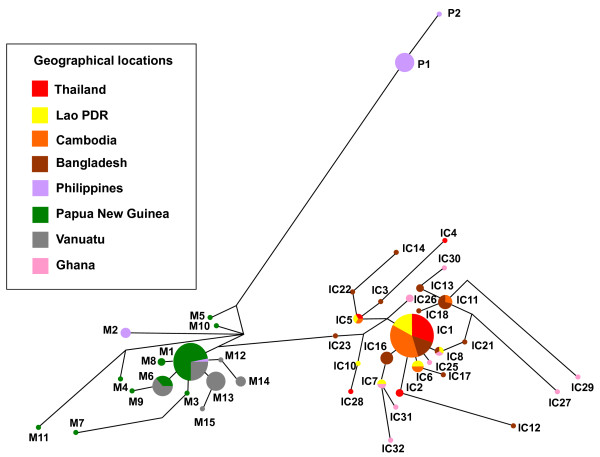
**Haplotype network diagram in *Plasmodium falciparum *isolates harbouring *pfcrt *mutation**. Network tree is shown according to countries where samples were taken. The haplotype network was constructed for 196 *P. falciparum *isolates harbouring *pfcrt *mutation comprises 49 microsatellite haplotypes, based on allelic variations in six microsatellite loci flanking the *pfcrt *locus (see text for details). The size of each circle corresponds to the number of samples sharing the same haplotype, and the length of an edge is proportional to a variation in repeat number between two haplotypes. IC; Indochina lineage, M; Melanesian lineage, P; Philippine lineage.

The geographical distribution of the three major lineages described above was clearly distinctive (Figure 4). The Indochina lineage was widely distributed in Indochina and Africa, and this is consistent with previous reports [1,11]. Distribution of the Melanesian lineage was limited to Papua New Guinea and Vanuatu, except for four isolates (M1 and M2 found in the Philippines, which probably migrated from Melanesia).

## Discussion

The present study using 256 *P. falciparum *isolates collected from Asia, Melanesia and Africa revealed only three major lineages of CQ resistance originating in Indochina, Melanesia and the Philippines. No novel *pfcrt *genotype was identified in the present samples collected from large-scale survey. The number of CQ-mutant lineages observed in this study was identical to that in two previous studies that analysed 48 laboratory-adapted CQ-resistant parasite lines [1] and field isolates in the Philippines [14]. This observation supports the hypothesis that the emergence of CQ resistance is a rare event, but the reasons for this rare emergence of CQ resistance remain to be clarified.

This study also revealed that a single CQ resistance lineage contained two mutant *pfcrt *genotypes; the CVIET and CVIDT genotypes in the Indochina lineage, and the SVMNT and CVMNT genotypes in the Melanesian lineage. Similar findings have been reported previously; two mutant genotypes (CVIET and CVIES) in a single CQ-resistant lineage in Africa [11] and two mutant genotypes (CVMNT and CVMET) in a single CQ-resistant lineage in South America [1]. In the present study, CVIET was the predominant mutant genotype in Asia and Africa, except in Lao P.D.R., where the CVIDT genotype was more prevalent than CVIET. A high prevalence of the CVIET genotype supports the notion that this genotype is an ancestral mutant genotype in the Indochina lineage, from which the CVIDT genotype may have evolved. Similarly, the CVMNT genotype, which was observed in only one case, appears to be a descendent of the SVMNT genotype of the Melanesian lineage. These additional amino acid changes may have been generated after the emergence of CQ resistance, perhaps in the last 30-40 years [37]. Thus, it is likely that additional amino acid change(s) at positions 72-75 in *pfcrt *were recently generated in parasites harbouring the *pfcrt *K76T mutation.

The role of *pfcrt *mutations other than K76T remains to be fully elucidated. They may confer some benefit to CQ-resistant parasites, e.g., a small effect on CQ tolerance or compensation for impaired protein activity after the acquisition of the critical mutation K76T. For the conventional anti-malarial drug pyrimethamine, it is known that levels of resistance increase as mutations progressively accumulate in *dhfr*; relative IC_50 _values of pyrimethamine for mutant *dhfr *genotypes, as compared to the wild *dhfr *genotype, are 35-fold higher in a single mutant and 1,111-fold higher in a quadruple mutant (highest resistance *dhfr *genotype) [38]. Similarly, association between the accumulation of mutations in *dhps *and progressive increases of sulphadoxine resistance have also been reported [39]. Further studies are necessary to clarify the potential association between the accumulation of additional *pfcrt *mutations and augmentation of CQ resistance.

The present analysis did not identify a back mutation at position 76 in *pfcrt*, as observed in previous studies [26-28]. However, there remains the possibility that the back mutation has occurred in geographic areas not studied here. Most of the isolates sampled in the present study came from Asia and Melanesia, where CQ is still being used for treatment of *Plasmodium vivax*. Thus, the continuous CQ pressure present in Asia and Melanesia may be suppressing the expression of this back mutation. Further molecular epidemiological studies in different endemic areas having different histories of CQ usage may be necessary in order to better understand the possible back mutation at the critical position 76, which would indicate the recovery of CQ susceptibility.

The conclusions of the present study are limited to samples collected from the late 1990s to the mid 2000s in geographical areas where samples were collected. Although the intercontinental migration of CQ resistance from Asia to Africa was already accomplished, the selection and spread of CQ resistance was not homogenously advanced in Asia. In fact, at the time of our sampling, while mutant *pfcrt *alleles were almost fixed in Thailand and Cambodia, the selection seemed to be ongoing in Lao PDR, where nearly half of *P. falciparum *still harboured the wild *pfcrt *genotype.

## Conclusions

The present molecular analysis using samples across Asia, Africa and Melanesia provided two important insights into CQ resistance in terms of malaria control. First, the emergence of novel CQ-mutant lineages among *P. falciparum *isolates is rare. Second, in isolates harbouring the K76T mutation, additional mutations in *pfcrt *other than K76T have readily been generated (in the last 30 years). These new mutations may confer stronger resistance to CQ than the K76T mutant. Further molecular monitoring of *pfcrt *genotypes should provide valuable information with regard to the current situation of CQ resistance and to the potential emergence of CQ-sensitive *P. falciparum *isolates in areas where CQ use has been withdrawn.

## Competing interests

The authors declare that they have no competing interests.

## Authors' contributions

NT performed experiments, data analysis and paper writing. TT1, MD, LD, BK, JS, MN, MS, JK, FH, TT2 and AK coordination of sampling. KT critically reviewed the manuscript. KT, HE and TM participated in acquisition of funding. TM made substantial contributions to study design, coordination of sampling, data analysis, paper writing and reviewing. All authors have read and approved the final manuscript.

## Supplementary Material

Additional file 1 **PCR conditions for genotyping of *pfcrt***. Click here for file

Additional file 2 **PCR conditions for determining variations of six microsatellite loci flanking *pfcrt***. Click here for file
